# Direct Comparison of Chol-siRNA Polyplexes and Chol-DsiRNA Polyplexes Targeting STAT3 in a Syngeneic Murine Model of TNBC

**DOI:** 10.3390/ncrna8010008

**Published:** 2022-01-13

**Authors:** Zhen Ye, Mai Mohamed Abdelmoaty, Stephen M. Curran, Shetty Ravi Dyavar, Devendra Kumar, Yazen Alnouti, Don W. Coulter, Anthony T. Podany, Rakesh K. Singh, Joseph A. Vetro

**Affiliations:** 1 Department of Pharmaceutical Sciences, College of Pharmacy, University of Nebraska Medical Center, Omaha, NE 68198, USA; yezhen1@gmail.com (Z.Y.); mai.mostafa@unmc.edu (M.M.A.); smcurran@unmc.edu (S.M.C.); devendra.kumar@unmc.edu (D.K.); yalnouti@unmc.edu (Y.A.); 2 Therapeutic Chemistry Department, Pharmaceutical and Drug Industries Research Division, National Research Centre, Giza 12622, Egypt; 3 Department of Pharmacy Practice, College of Pharmacy, University of Nebraska Medical Center, Omaha, NE 68198, USA; dyavarsravi@gmail.com (S.R.D.); apodany@unmc.edu (A.T.P.); 4 Center for Drug Delivery and Nanomedicine, University of Nebraska Medical Center, Omaha, NE 68198, USA; rsingh@unmc.edu; 5 Department of Pediatrics, Division of Pediatric Hematology/Oncology, University of Nebraska Medical Center, Omaha, NE 68198, USA; dwcoulter@unmc.edu; 6 Department of Pathology and Microbiology, University of Nebraska Medical Center, Omaha, NE 68198, USA

**Keywords:** RNAi, drug delivery, siRNA delivery, DsiRNA delivery, RNAi delivery, Chol-DsiRNA polymer micelles, Chol-siRNA polymer micelles

## Abstract

RNA interference (RNAi) molecules have tremendous potential for cancer therapy but are limited by insufficient potency after intravenous (IV) administration. We previously found that polymer complexes (polyplexes) formed between 3′-cholesterol-modified siRNA (Chol-siRNA) or DsiRNA (Chol-DsiRNA) and the cationic diblock copolymer PLL[30]-PEG[5K] greatly increase RNAi potency against stably expressed LUC mRNA in primary syngeneic murine breast tumors after daily IV dosing. Chol-DsiRNA polyplexes, however, maintain LUC mRNA suppression for ~48 h longer after the final dose than Chol-siRNA polyplexes, which suggests that they are the better candidate formulation. Here, we directly compared the activities of Chol-siRNA polyplexes and Chol-DsiRNA polyplexes in primary murine 4T1 breast tumors against STAT3, a therapeutically relevant target gene that is overexpressed in many solid tumors, including breast cancer. We found that Chol-siSTAT3 polyplexes suppressed STAT3 mRNA in 4T1 tumors with similar potency (half-maximal ED_50_ 0.3 mg/kg) and kinetics (over 96 h) as Chol-DsiSTAT3 polyplexes, but with slightly lower activity against total Stat3 protein (29% vs. 42% suppression) and tumor growth (11.5% vs. 8.6% rate-based T/C ratio) after repeated IV administration of equimolar, tumor-saturating doses every other day. Thus, both Chol-siRNA polyplexes and Chol-DsiRNA polyplexes may be suitable clinical candidates for the RNAi therapy of breast cancer and other solid tumors.

## 1. Introduction

RNA interference (RNAi) is a natural, intracellular process that selectively decreases the expression of a specific protein at the mRNA level through complementary base-pair binding of gene-specific dsRNA molecules, including microRNA (miRNA); small, interfering RNA (siRNA); or dicer-substrate siRNA (DsiRNA or “RNAi molecules”) [1,2]. Several proteins have been identified in tumor or tumor-associated cells where suppression may produce a therapeutic effect and/or increase the efficacy of other cancer treatments [2,3]. Thus, RNAi molecules have tremendous potential for improving cancer therapy.

**Figure 1:**
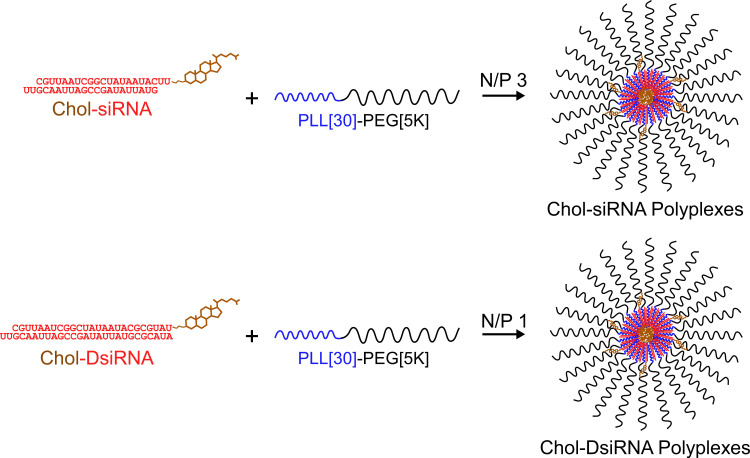
Idealized electrostatic self-assembly of Chol-siRNA and Chol-DsiRNA polyplexes formed with PLL[30]-PEG[5K]. A solution of negatively charged siRNA or DsiRNA (red) modified with 3′-cholesterol on the sense strand (brown) is added to a solution of positively charged diblock copolymers composed of 30 poly-L-lysine residue blocks (blue) and 5 kDa polyethylene glycol blocks (black) (PLL[30]-PEG[5K]) at the indicated N/P charge molar ratio of: mole(s) positively charged primary amines (N)/mole negatively charged phosphates (P). The negatively charged phosphate backbones of Chol-siRNA or Chol-DsiRNA then electrostatically bind and neutralize the positively charged PLL[30] blocks. This converts the PLL[30]-PEG[5K] unimers into amphiphilic diblock copolymers that spontaneously self-assemble into Chol-siRNA or Chol-DsiRNA polymer complexes (polyplexes) that are further stabilized by hydrophobic interactions between 3′-cholesterol groups within the polyplex core. Figure adapted and modified from [2].

Most cancer therapies require IV. administration to reach diffuse or inaccessible tumors [4]. The potencies of RNAi molecules after IV administration, however, are extremely low or undetectable [1]. Several types of nanoscale dosage forms have been developed to increase the potency of RNAi molecules after IV administration using a wide range of modified RNAi molecules and/or materials [2,5,6,7]. We previously found that electrostatically self-assembled polymer complexes (polyplexes), composed of siRNA or DsiRNA modified by 3′-cholesterol on the sense strand (Chol-siRNA/Chol-DsiRNA) and a diblock copolymer of 30 poly-L-lysine residues and 5 kDa polyethylene glycol (PLL[30]-PEG[5K]) (Figure 1), greatly increase RNAi potency against stably expressed LUC mRNA in primary murine 4T1-Luc breast tumors after daily IV administration of tumor-saturating doses [2.5 mg Chol-RNAi/kg] over three days without affecting body weight [4,8]. Chol-DsiRNA polyplexes, however, maintain LUC mRNA suppression ~48 h longer after the final dose than Chol-siRNA polyplexes [4,8], have higher loading (50 wt% vs. 25 wt%), provide greater protection against RNase degradation in 90% murine serum [8], and are well tolerated by healthy female BALB/c mice to at least 50 mg Chol-DsiRNA/kg after repeated IV administration over 28 days [2]. This suggests Chol-DsiRNA polyplexes are a more promising formulation candidate than Chol-siRNA polyplexes for the RNAi therapy of breast cancer and possibly other solid tumors.

In contrast to the suppression of stably expressed LUC mRNA in 4T1-Luc tumors, we recently found that Chol-DsiRNA polyplexes targeting STAT3, a more widely expressed and therapeutically relevant mRNA, maintain suppression of STAT3 mRNA in primary 4T1 breast tumors less than 24 h after a single lower dose of Chol-DsiSTAT3 [0.5 mg/kg] that does not saturate the primary tumor [2]. Furthermore, the activity of Chol-siRNA polyplexes against STAT3 mRNA in primary 4T1 breast tumors has not been determined or as thoroughly characterized as Chol-DsiRNA polyplexes. Thus, it remains unclear whether there are also differences in the activities of Chol-siRNA polyplexes and Chol-DsiRNA polyplexes against therapeutically relevant mRNA in solid breast tumors. In this study, we address possible differences in polyplex activities against therapeutically relevant mRNA in solid breast tumors by directly comparing the activities of Chol-siRNA polyplexes and Chol-DsiRNA polyplexes targeting STAT3 expression in primary murine 4T1 breast tumors after IV administration.

## 2. Results

### 2.1. Activities of 5′-Overlapping siSTAT3 and DsiSTAT3 Sequences in Murine Syngeneic 4T1 Breast Cancer Epithelial Cells

Differences in the activities of Chol-siRNA polyplexes and Chol-DsiRNA polyplexes after IV administration may be due, in part, to differences in the activities of the respective siRNA and DsiRNA sequences in the target cell. We previously found that electroporation with equimolar concentrations of a siLUC sequence or 5′-overlapping DsiLUC sequence (i.e., a 3′-extension of the siLUC sequence) suppresses stably expressed LUC mRNA in 4T1-Luc cells to the same extent and duration over 72 h [8]. As such, we expected that the 5′-overlapping siSTAT3 and DsiSTAT3 sequences would have similar activities against murine STAT3 mRNA in 4T1 cells.

**Figure 2:**
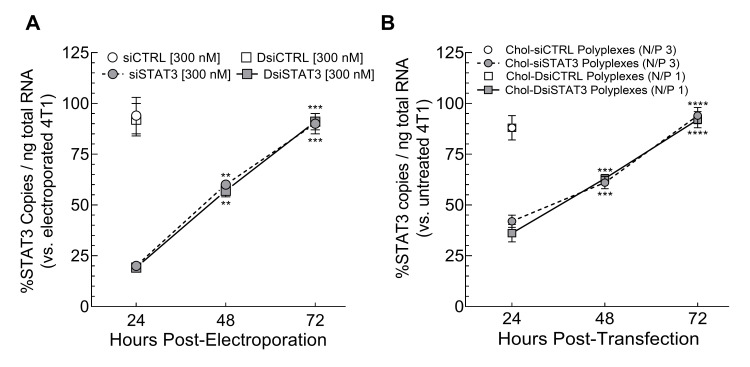
Activities of 5′-overlapping siSTAT3 and DsiSTAT3 sequences and corresponding Chol-siSTAT3 polyplexes and Chol-DsiSTAT3 polyplexes in murine 4T1 breast cancer epithelial cells over 72 h. Murine 4T1 cells were (**A**) electroporated alone or in the presence of inactive siCTRL (white circle), inactive DsiCTRL (white square), siSTAT3 (grey circles, dashed line), or 5′-overlapping DsiSTAT3 (grey squares, solid line) [300 nM] and then incubated at 37 °C or (**B**) incubated for 4 h with serum-free media alone or containing inactive Chol-siCTRL (white circle), inactive Chol-DsiCTRL (white square), Chol-siSTAT3 (grey circles, dashed line), or 5′-overlapping Chol-DsiSTAT3 (grey squares, solid line) [200 nM], complexed with PLL[30]-PEG[5K] at the indicated N/P ratio, before adding media containing 20% FBS at 1/1 (*v*/*v*) and incubating at 37 °C. Average murine STAT3 mRNA copy numbers per ng total RNA ± propagated SD *n* = 2 (**A**) or 3 (**B**) independent treatments were determined at the indicated time points by RT-ddPCR (Table S1) and normalized to either electroporation-only 4T1 cells (**A**) or untreated 4T1 cells (**B**) at the same time point, respectively. Averages were then compared at each time point to either unpaired two-tailed *t*-test (no significant differences observed) or to the 24-h time point by ordinary one-way ANOVA with Dunnett’s post-test, where ** *p* < 0.01, *** *p* < 0.001, and **** *p* < 0.0001 (siSTAT3 above symbols/DsiSTAT3 below symbols). Results are representative of at least two independent experiments. Data for DsiCTRL, DsiSTAT3, Chol-DsiCTRL polyplexes, and Chol-DsiSTAT3 polyplexes are taken from [2].

To determine whether the activities of the 5′-overlapping siSTAT3 and DsiSTAT3 sequences against STAT3 mRNA are similar in murine syngeneic breast cancer epithelial cells, we electroporated 4T1 cells with equimolar concentrations of siSTAT3, DsiSTAT3 (a 3′-extension of the siSTAT3 sequence that suppresses 81% of murine STAT3 mRNA in 4T1 cells 24 h after electroporation at 300 nM [2]), inactive siCTRL, or inactive DsiCTRL and compared normalized murine STAT3 mRNA copy numbers to electroporated 4T1 cells over 72 h with RT-ddPCR (Figure 2A). Electroporation with siSTAT3 (Figure 2A, grey circles, dashed line) decreased STAT3 mRNA copy numbers to the same extent as electroporation with DsiSTAT3 (Figure 2A, grey squares, solid line) at 24, 48, and 72 h. In contrast, electroporation with siCTRL (Figure 2A, white circle) or DsiCTRL (Figure 2A, white square) had no effect after 24 h, indicating that suppression of murine STAT3 mRNA is due to the respective siSTAT3 and DsiSTAT3 sequences. Thus, the activities of the current 5′-overlapping siSTAT3 and DsiSTAT3 sequences are similar in syngeneic breast cancer epithelial cells over 72 h.

### 2.2. Hydrodynamic Diameters, Surface Charges, and Physical Stabilities of Chol-siRNA Polyplexes and Chol-DsiRNA Polyplexes

Polyplex diameter and surface charge are two physicochemical properties that can potentially affect the activity of polyplexes after IV administration [9,10]. To determine whether there are differences in the diameters and/or surface charges of Chol-siRNA polyplexes and Chol-DsiRNA polyplexes, we compared the average hydrodynamic diameters and zeta potentials of Chol-siCTRL polyplexes and Chol-DsiCTRL polyplexes in the complexation buffer (0.1 M HEPES, pH 7.4) with nanoparticle tracking analysis and DLS, respectively (Table 1). We used the respective N/P ratios of PLL[30]-PEG[5K] that greatly slow Chol-siRNA and Chol-DsiRNA migration in agarose gels (Figure S1, Lanes 2 & 4) [4,8], protect complexed Chol-siRNA and Chol-DsiRNA against nuclease degradation in 90% (*v*/*v*) murine serum [4,8], and form active polyplexes in vitro and in vivo [2,4,8].

Chol-siCTRL polyplexes had a slightly smaller hydrodynamic diameter than Chol-DsiCTRL polyplexes 30 min after complexation [25 ± 2 (SD) vs. 33 ± 2 nm, *p* = 0.0080, *n* = 3 independent analyses] but had a similar zeta potential [8 ± 2 (SD) vs. 5.2 ± 0.7 mV, *p* = 0.0840, *n* = 3 independent analyses] (Table 1). The hydrodynamic diameter of Chol-siSTAT3 polyplexes, however, began to increase between 3 h and 6 h, whereas the hydrodynamic diameter of Chol-DsiSTAT3 polyplexes was constant over 24 h (Figure S3). Thus, Chol-siRNA polyplexes have a slightly smaller hydrodynamic diameter and similar surface charge compared to Chol-DsiRNA polyplexes but are less physically stable in solution over 24 h under the current formulation conditions.

### 2.3. Activities of Chol-siSTAT3 Polyplexes and Chol-DsiSTAT3 Polyplexes in Murine Syngeneic 4T1 Breast Cancer Epithelial Cells

Differences in the activities of Chol-siRNA polyplexes and Chol-DsiRNA polyplexes in primary tumors after IV administration may be due, in part, to differences in their respective activities within the target cell. To determine whether there are differences between the activities of Chol-siRNA polyplexes and Chol-DsiRNA polyplexes against STAT3 mRNA in syngeneic murine breast cancer epithelial cells, we transfected 4T1 cells with equimolar concentrations of the corresponding Chol-siCTRL, Chol-DsiCTRL, Chol-siSTAT3, or Chol-DsiSTAT3 sequences (Figure 2A) complexed with PLL[30]-PEG[5K] at the indicated N/P ratio and compared normalized murine STAT3 mRNA copy numbers to untreated 4T1 cells 24, 48, and 72 h after treatment by RT-ddPCR (Figure 2B). Transfection with Chol-siSTAT3 polyplexes (Figure 2B, grey circles, dashed line) decreased STAT3 mRNA copy numbers below untreated 4T1 cells to the same extent as Chol-DsiSTAT3 polyplexes (Figure 2B, grey squares, solid line) at each time point. In contrast, transfection with Chol-siCTRL polyplexes (Figure 2B, white circle) or Chol-DsiCTRL polyplexes (Figure 2B, white square) decreased STAT3 mRNA copy numbers by 12% at 24 h, indicating that STAT3 mRNA suppression by Chol-siSTAT3 or Chol-DsiSTAT3 polyplexes is primarily due to the activities of complexed Chol-siSTAT3 or Chol-DsiSTAT3, respectively. Thus, the activities of the current polyplexes are similar in syngeneic murine breast cancer epithelial cells over 72 h at the current N/P ratios.

### 2.4. Potencies of Chol-siSTAT3 Polyplexes and Chol-DsiSTAT3 Polyplexes in Primary Murine Syngeneic Breast Tumors after IV Administration

The 4T1 syngeneic breast cancer epithelial cell line is a good murine model for TNBC because it lacks ER, PR, and HER2; can be grown in immune competent female BALB/c mice; is poorly immunogenic; shares substantial molecular features with human TNBC [11]; has rates of growth and metastatic patterns that resemble human breast cancer; and presents late-stage disease that is comparable to stage IV breast cancer [12,13]. STAT3 expression is also essential for primary 4T1 tumor growth and metastasis [14].

**Figure 3:**
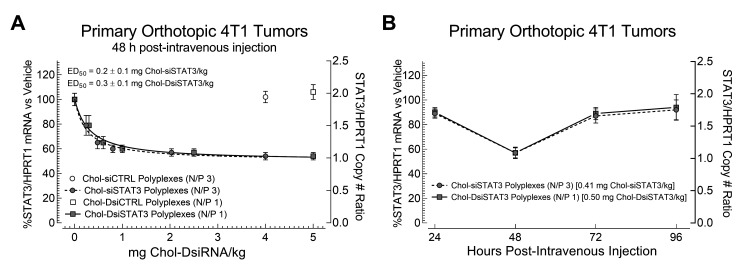
Dose response and kinetics of STAT3 mRNA suppression in primary syngeneic murine breast tumors by Chol-siSTAT3 polyplexes and Chol-DsiSTAT3 polyplexes after IV administration. (**A**) Vehicle alone (HEPES/0.15 M NaCl; 0.1 mL) or vehicle containing the indicated dose of Chol-siSTAT3 (grey circles, dashed line), Chol-DsiSTAT3 (grey squares, solid line), inactive Chol-siCTRL (white circle), or inactive Chol-DsiCTRL (white square) complexed with PLL[30]-PEG[5K] at the indicated N/P ratio was injected into the tail veins of female BALB/c mice bearing a single subcutaneous 4T1 breast tumor (~30 to 50 mm^3^). After 48 h, average ratios of murine STAT3 to murine HPRT1 mRNA copy numbers in primary 4T1 breast tumors ± propagated SD (*n* = 5 mice) were determined by RT-ddPCR (Table S2) and half-maximal ED_50_ values were calculated for each polyplex from nonlinear fits of their respective dose-response curves. (**B**) Vehicle alone (HEPES/0.15 M NaCl; 0.1 mL) or vehicle containing an equimolar dose of Chol-siSTAT3 (grey circles, dashed line) or Chol-DsiSTAT3 (grey squares, solid line) complexed with PLL[30]-PEG[5K] at the indicated N/P ratio was injected into the tail veins of female BALB/c mice bearing a single subcutaneous 4T1 breast tumor (~30 to 50 mm^3^). At the indicated time points after injection, average ratios of murine STAT3 to murine HPRT1 mRNA copy numbers in primary 4T1 breast tumors ± propagated SD (*n* = 5 mice) were determined by RT-ddPCR then compared at the same time point by two-tailed Mann–Whitney test. Data for vehicle, Chol-DsiCTRL polyplexes, and Chol-DsiSTAT3 polyplexes are taken from [2]. Results are representative of at least two independent experiments.

To determine potential differences between the potencies and efficacies of Chol-siRNA polyplexes and Chol-DsiRNA polyplexes against STAT3 mRNA in primary murine syngeneic breast tumors after IV administration, we intravenously administered increasing equimolar doses of Chol-siSTAT3 or Chol-DsiSTAT3 or a single maximum equimolar dose of inactive Chol-siCTRL or inactive Chol-DsiCTRL complexed with PLL[30]-PEG[5K] at the indicated N/P ratio and compared normalized murine STAT3 mRNA copy numbers in early-stage primary 4T1 breast tumors to vehicle alone by RT-ddPCR (Figure 3A). We chose a 48-h timepoint given that Chol-DsiSTAT3 polyplexes maximally suppress STAT3 mRNA in primary murine 4T1 breast tumors 48 h after IV administration [2].

Chol-siSTAT3 polyplexes (Figure 3A, grey circles) suppressed STAT3 mRNA copy numbers in primary 4T1 breast tumors to a similar extent as Chol-DsiSTAT3 polyplexes (Figure 3A, grey squares) at the highest equimolar dose [47 ± 5 (SD) vs. 46 ± 3%] with a similar half-maximal ED_50_ [0.2 Chol-siSTAT3 ± 0.1 (SD) vs. 0.3 Chol-DsiSTAT3 ± 0.1 mg/kg] (Figure 3A, dashed line vs. solid line). In contrast, inactive Chol-siCTRL polyplexes (Figure 3A, white circle) or inactive Chol-DsiCTRL polyplexes (Figure 3A, white square) had no effect on STAT3 mRNA levels at the highest equimolar doses of Chol-siSTAT3 or Chol-DsiSTAT3, indicating that STAT3 mRNA suppression by Chol-siSTAT3 polyplexes and Chol-DsiSTAT3 polyplexes is due to the activities of complexed Chol-siSTAT3 or Chol-DsiSTAT3, respectively. Thus, Chol-siRNA polyplexes and Chol-DsiRNA polyplexes have similar potencies and efficacies against STAT3 mRNA in primary murine syngeneic breast tumors after IV administration at the current N/P ratios.

### 2.5. Kinetics of mRNA Suppression in Primary Murine Syngeneic Breast Tumors by Chol-siSTAT3 Polyplexes and Chol-DsiSTAT3 Polyplexes after IV Administration

To determine whether there are differences between the kinetics of STAT3 mRNA suppression in primary murine syngeneic breast tumors by Chol-siSTAT3 and Chol-DsiSTAT3 polyplexes after IV administration, we intravenously administered a single equimolar dose of Chol-siSTAT3 or Chol-DsiSTAT3 complexed with PLL[30]-PEG[5K] at the indicated N/P ratio and compared normalized STAT3 mRNA copy numbers in early-stage primary murine 4T1 breast tumors to vehicle alone every 24 h over 96 h by RT-ddPCR (Figure 3B). We chose an equimolar dose of Chol-siSTAT3 [0.41 mg/kg] and Chol-DsiSTAT3 [0.5 mg/kg] that does not maximally suppress STAT3 mRNA in primary 4T1 breast tumors (Figure 3A) to eliminate possible effects of tumor dose saturation on the kinetics of mRNA suppression in the primary tumor. Chol-siSTAT3 polyplexes (Figure 3B, grey circles, dashed line) suppressed STAT3 mRNA copy numbers in primary 4T1 breast tumors to a similar extent as Chol-DsiSTAT3 polyplexes (Figure 3B, grey squares, solid line) at 24 h [11 ± 4 (SD) vs. 10 ± 4%], 48 h [43 ± 4 (SD) vs. 43 ± 5%], 72 h [13 ± 6 (SD) vs. 11 ± 5%], and 96 h [8 ± 8 (SD) vs. 6 ± 10%]. Thus, Chol-siSTAT3 polyplexes and Chol-DsiSTAT3 polyplexes suppress STAT3 mRNA in primary murine syngeneic breast tumors with similar kinetics after IV administration below tumor-saturating doses at the current N/P ratios.

### 2.6. Chol-siSTAT3 Polyplex and Chol-DsiSTAT3 Polyplex Activities against the Growth of Primary Murine Syngeneic Breast Tumors after IV Administration

**Figure 4:**
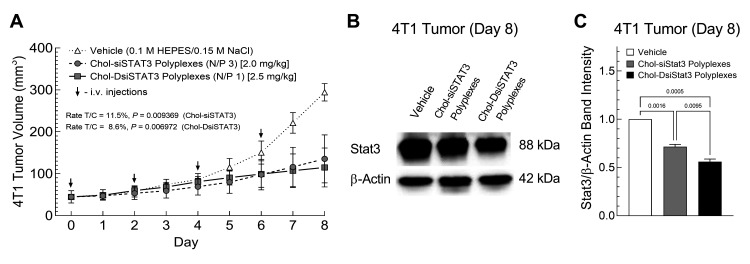
Effect of Chol-siSTAT3 polyplexes and Chol-DsiSTAT3 polyplexes on primary syngeneic murine breast tumor growth and STAT3 protein expression after multiple IV treatments. (**A**) Vehicle alone (white triangles) or vehicle containing equimolar doses of inactive Chol-siCTRL (Figure S4, white circles), inactive Chol-DsiCTRL (Figure S4, white squares), Chol-siSTAT3 (grey circles), or Chol-DsiSTAT3 (grey squares) complexed with PLL[30]-PEG[5K] at the indicated N/P ratio was injected into the tail veins of 4- to 6-week-old female BALB/c mice (black arrows) bearing a single subcutaneous 4T1 breast tumor (~30 to 50 mm^3^). Average daily tumor volumes ±SD (*n* = 5 mice) were then determined by 3D surface scanning, compared at each time point by multiple Mann–Whitney tests (polyplexes), and a rate-based T/C ratio with associated *p* value was calculated for each polyplex (**B**) On Day 8 (48 h after final IV injection), steady-state levels of murine STAT3 and murine β-Actin protein in primary 4T1 tumors were determined by western blot, and then (**C**) average ratios of Stat3/β-Actin protein band intensities ±SD (*n* = 2 measurements) were determined by imaging densitometry and compared by ordinary one-way ANOVA. Protein bands from images of the same western blot were used to generate (**B**). Data for vehicle, Chol-DsiCTRL, and Chol-DsiSTAT3 are taken from [2]. Results are representative of at least two independent experiments.

STAT3 is critical for the growth of murine 4T1 breast tumors [14,15,16]. Thus, to determine whether there are differences in the therapeutic activities of Chol-siSTAT3 Polyplexes and Chol-DsiSTAT3 polyplexes against primary murine syngeneic breast tumors after IV administration, we intravenously administered an equimolar dose of Chol-siSTAT3, Chol-DsiSTAT3, inactive Chol-siCTRL, or inactive Chol-DsiCTRL complexed with PLL[30]-PEG[5K] at the indicated N/P ratio every other day to female BALB/c mice bearing early-stage 4T1 breast tumors and compared tumor volumes to vehicle alone over 8 days by 3D surface scanning (Figure 4A and Figure S2). We chose the lowest equimolar dose of Chol-siSTAT3 [2 mg/kg] and Chol-DsiSTAT3 [2.5 mg/kg] that provided maximum suppression of STAT3 mRNA levels in primary 4T1 breast tumors (Figure 3A) to minimize possible effects of dose saturation in the primary tumor.

Chol-siSTAT3 polyplexes (Figure 4A, grey circles, dashed line) inhibited the growth of primary 4T1 tumors at a slightly higher rate-based T/C ratio [17] than Chol-DsiSTAT3 polyplexes (Figure 4A, grey squares, solid line) vs. vehicle alone (Figure 4A, white triangles) [11.5% vs. 8.6%] where T/C ratios ≤10% indicate high anti-tumor activity at non-toxic doses [17]. In contrast, Chol-siCTRL polyplexes (Figure S4, white circles) or Chol-DsiCTRL polyplexes (Figure S4, white squares) did not affect the growth of primary murine 4T1 breast tumors vs. vehicle alone (Figure S4, white triangles), indicating that the inhibition of 4T1 tumor growth was due to the activities of complexed Chol-siSTAT3 or Chol-DsiSTAT3, respectively. Consistent with slightly lower therapeutic activity against primary 4T1 breast tumor growth (Figure 4A), Chol-siSTAT3 polyplexes (Figure 4C, grey bar) suppressed 13% less total Stat3 protein in the primary 4T1 tumors than Chol-DsiSTAT3 polyplexes (Figure 4C, black bar) vs. vehicle alone 48 h after the final IV dose (Day 8) [29 ± 2 (SD) vs. 42 ± 2% suppression]. Furthermore, average body weights of mice from all treatment groups gradually increased over the course of the study (not shown), indicating that: (i.) Chol-siSTAT3 and Chol-DsiSTAT3 polyplexes are not toxic under the current dosage regimen, and (ii.) tumor growth inhibition is due to the activity of complexed Chol-siSTAT3 or Chol-DsiSTAT3 and not abnormal weight loss. Thus, Chol-siSTAT3 polyplexes have slightly lower therapeutic activity against primary murine syngeneic breast tumors than Chol-DsiSTAT3 polyplexes with the current dosage regimen and N/P ratios.

### 2.7. Pharmacokinetics of Chol-DsiSTAT3 and Chol-DsiSTAT3 Polyplexes in Healthy Female BALB/c Mice after IV Administration

**Figure 5:**
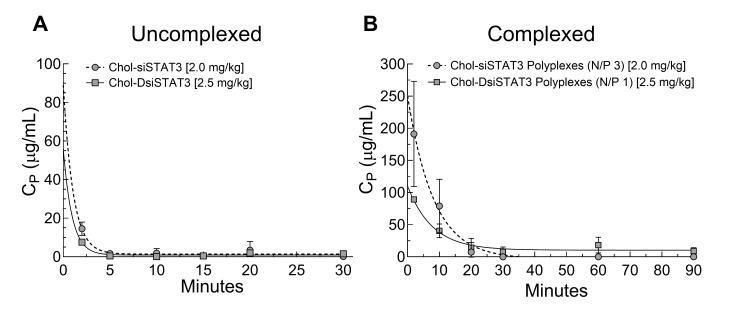
Pharmacokinetics of complexed Chol-siSTAT3 and Chol-DsiSTAT3 in healthy young female BALB/c mice after IV administration. An equimolar dose of Chol-siSTAT3 [2.0 mg/kg] (black circles) or Chol-DsiSTAT3 [2.5 mg/kg] (grey squares) (**A**) alone or (**B**) complexed with PLL[30]-PEG[5K] at the indicated N/P ratio was injected into the tail veins of healthy female BALB/c mice (4 to 6 weeks old) and average plasma concentrations of Chol-siSTAT3 or Chol-DsiSTAT3 ± SD (*n* = 5 mice), respectively, were determined indirectly at each time point as differences in total extracted RNA from the plasma of treated vs. untreated mice by fluorescence assay. Error bars are present in (**A**) but sometimes indistinguishable at the current scale. Data for Chol-DsiSTAT3 and Chol-DsiSTAT3 polyplexes taken from [2].

To next determine whether there are differences between the pharmacokinetics of complexed Chol-siRNA and Chol-DsiRNA, we intravenously administered equimolar doses of Chol-siSTAT3 or Chol-DsiSTAT3 alone or complexed with PLL[30]-PEG[5K] at the indicated N/P ratio into young healthy female BALB/c mice. Given that total RNA in the plasma of untreated mice is relatively constant (not shown), we then indirectly determined plasma concentrations of Chol-siSTAT3 or Chol-DsiSTAT3 over time as an increase in the total RNA extracted from the plasma of treated vs. untreated mice (Figure 5) for pharmacokinetic analysis (Table 2).

The high variability of plasma concentrations and limited number of time points (Figure 5A,B) prevented complete and accurate compartmental analysis of the PK profiles. Despite the limited analysis, uncomplexed Chol-siSTAT3 and Chol-DsiSTAT3 had a statistically similar AUC [2.3 ± 0.9 (SD) vs. 3 ± 1 h·μg/mL, *p* = 0.2782] and mean residence time [0.07 ± 0.03 (SD) vs. 0.8 ± 0.9 h, *p* = 0.1074] (Table 2). Although Chol-siSTAT3 polyplexes increased the AUC [19 ± 2 (propagated SD) vs. 13 ± 2-fold increase in AUC, *p* <0.0021] and mean residence time [7.1 ± 0.5 (propagated SD) vs. 1 ± 1-fold increase in MRT, *p* < 0.0001] vs. uncomplexed Chol-siSTAT3 to a greater extent than Chol-DsiSTAT3 polyplexes vs. uncomplexed Chol-DsiSTAT3 (Table 2), complexed Chol-siSTAT3 and Chol-DsiSTAT3 also had a similar AUC [44 ± 2 (SD) vs. 39 ± 13 h·μg/mL, *p* = 0.4200] and mean residence time [0.5 ± 0.5 (SD) vs. 0.9 ± 0.4 h, *p* = 0.2000] (Table 2). Thus, Chol-siSTAT3 polyplexes and Chol-DsiSTAT3 polyplexes increase plasma exposure and mean residence time of complexed Chol-siSTAT3 and Chol-DsiSTAT3 to similar levels in young, healthy female BALB/c mice at the current N/P ratios.

### 2.8. Distribution of Complexed and Uncomplexed Chol-siSTAT3 and Chol-DsiSTAT3 in 4T1 Breast Tumor-Bearing Female BALB/c Mice

**Figure 6:**
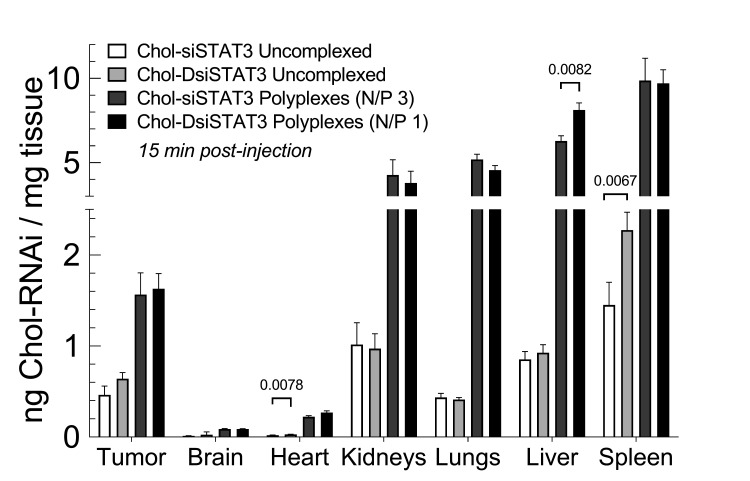
Effect of Chol-siRNA polyplexes and Chol-DsiRNA polyplexes on the distribution of Chol-siSTAT3 and Chol-DsiSTAT3 in syngeneic 4T1 breast tumor-bearing mice after IV administration. An equimolar dose of Chol-siSTAT3 [2.0 mg/kg] or Chol-DsiSTAT3 [2.5 mg/kg] alone or complexed with PLL[30]-PEG[5K] at the indicated N/P ratio was injected intravenously into female BALB/c mice bearing a single 4T1 breast tumor (~30 to 50 mm^3^). After 15 min, average ng of Chol-DsiSTAT3/mg tissue ± propagated SD (*n* = 5 mice) was determined by RT-ddPCR of the respective antisense strands and compared (uncomplexed vs. uncomplexed; polyplex vs. polyplex) in each tissue by unpaired two-tailed *t*-tests. The *Y*-axis was truncated to facilitate comparison among treatment groups. Data for Chol-DsiSTAT3 and Chol-DsiSTAT3 polyplexes are taken from [2].

To next determine whether there are differences in the distributions of complexed Chol-siSTAT3 and Chol-DsiSTAT3 after IV administration, we intravenously administered an equimolar dose of Chol-siSTAT3 or Chol-DsiSTAT3 alone or complexed with PLL[30]-PEG[5K] at the indicated N/P ratio into young 4T1 breast tumor-bearing female BALB/c mice and compared the distributions of Chol-siSTAT3 or Chol-DsiSTAT3 in perfused organs and 4T1 breast tumors fifteen minutes post-injection by RT-ddPCR of the anti-sense strand (Figure 6). We chose a fifteen-minute time point to minimize RNase degradation of the distributed Chol-siRNA and Chol-DsiRNA molecules [7].

Uncomplexed Chol-siSTAT3 had a similar distribution to uncomplexed Chol-DsiSTAT3 in the tumor [0.46 ± 0.1 (SD) vs. 0.6 ± 0.2 ng/mg tissue, *p* = 0.0588], brain [0.013 ± 0.001 (SD) vs. 0.03 ± 0.07 ng/mg tissue, *p* = 0.6890], kidneys [1.0 ± 0.2 (SD) vs. 1.0 ± 0.4 ng/mg tissue, *p* = 0.8206], lungs [0.43 ± 0.05 (SD) vs. 0.41 ± 0.05 ng/mg tissue, *p* = 0.4680], and liver [0.85 ± 0.09 (SD) vs. 0.9 ± 0.2 ng/mg, *p* = 0.4692] but had a slightly lower distribution than uncomplexed Chol-DsiSTAT3 in the heart [0.020 ± 0.003 (SD) vs. 0.029 ± 0.005 ng/mg, *p* = 0.0078] and spleen [1.4 ± 0.2 (SD) vs. 2.3 ± 0.4 ng/mg, *p* = 0.0067] (Figure 6).

Chol-siSTAT3 polyplexes, except for a greater increase in the spleen [7 ± 2 (SD) vs. 4 ± 1 ng/mg tissue increase vs. uncomplexed Chol-RNAi molecule, *p* = 0.0171], increased the distribution of complexed Chol-siSTAT3 vs. uncomplexed Chol-siSTAT3 to a similar extent as Chol-DsiSTAT3 polyplexes vs. uncomplexed Chol-DsiSTAT3 in the tumor, brain, heart, kidneys, lungs, and liver (Figure 6). Complexed Chol-siSTAT3 also had a similar distribution as complexed Chol-DsiSTAT3 in the tumor [1.6 ± 0.5 (SD) vs. 1.6 ± 0.4 ng/mg tissue, *p* = 0.8294], brain [0.09 ± 0.01 (SD) vs. 0.088 ± 0.007 ng/mg tissue, *p* = 0.9557], heart [0.22 ± 0.03 (SD) vs. 0.27 ± 0.04 ng/mg tissue, *p* = 0.0828], kidneys [4 ± 2 (SD) vs. 4 ± 2 ng/mg tissue, *p* = 0.6902], lungs [5.2 ± 0.7 (SD) vs. 4.6 ± 0.6 ng/mg tissue, *p* = 0.1693], and spleen [10 ± 3 (SD) vs. 10 ± 2 ng/mg tissue, *p* = 0.9178] but had a slightly lower distribution in the liver [6.3 ± 0.7 (SD) vs. 8.1 ± 0.9 ng/mg tissue, *p* = 0.0082]. Thus, except for the liver, Chol-siRNA polyplexes and Chol-DsiRNA polyplexes increase the distribution of complexed Chol-siSTAT3 and Chol-DsiSTAT3, respectively, to a similar extent in syngeneic breast tumors and other organs in female BALB/c mice after IV administration at the current N/P ratios.

## 3. Discussion

We previously found that Chol-DsiLUC polyplexes maintain the suppression of stably expressed LUC mRNA in primary murine 4T1-Luc breast tumors for ~48 h longer after the final dose than Chol-siLUC polyplexes [8]. More recently, we found that Chol-DsiSTAT3 polyplexes suppress endogenous, therapeutically relevant STAT3 mRNA in primary 4T1 breast tumors less than 24 h after the final dose [2]. Thus, it remained unclear whether Chol-DsiRNA polyplexes also maintain suppression of endogenous, therapeutically relevant mRNA in solid breast tumors for a longer duration than Chol-siRNA polyplexes.

Our current study provides evidence that Chol-siRNA polyplexes suppress an endogenous, therapeutically relevant mRNA in early-stage primary murine 4T1 breast tumors with similar potency, efficacy, and kinetics as Chol-DsiRNA polyplexes but slightly lower therapeutic activity at the current N/P ratios. We found that Chol-siSTAT3 polyplexes (N/P 3) and Chol-DsiSTAT3 polyplexes (N/P 1) suppressed STAT3 mRNA in primary murine 4T1 breast tumors (~30 to 50 mm^3^) with similar half-maximal ED_50_ [0.2 ± 0.1 mg Chol-siSTAT3/kg vs. 0.3 ± 0.1 mg Chol-DsiSTAT3/kg] (Figure 3A), maximal suppression [47%] (Figure 3A), and kinetics over 96 h (Figure 3B), whereas Chol-siSTAT3 polyplexes suppressed slightly lower levels of total Stat3 protein [29% vs. 42%] (Figure 4C) and 4T1 tumor growth [rate-based T/C ratio of 11.5% vs. 8.6%] (Figure 4A) after repeated IV administration every other day over 6 days.

Our study also provides evidence that Chol-siSTAT3 polyplexes and Chol-DsiSTAT3 polyplexes increase plasma exposure of complexed Chol-RNAi molecules in healthy, young female BALB/c mice and subsequent localization to primary murine syngeneic breast tumors to similar levels after IV administration at the current N/P ratios. We found that Chol-siRNA polyplexes and Chol-DsiRNA polyplexes: (i.) increased the AUC [44 ± 2 (SD) vs. 39 ± 13 h·μg/mL, *p* = 0.4200] and mean residence time [0.5 ± 0.5 (SD) vs. 0.9 ± 0.4 h, *p* = 0.2000] of complexed Chol-siSTAT3 and Chol-DsiSTAT3, respectively, to similar levels in healthy female BALB/c mice (Table 2), and (ii.) increased the distribution of complexed Chol-siSTAT3 and Chol-DsiSTAT3, respectively, to similar levels in primary 4T1 breast tumors 15 min after IV administration [1.6 ± 0.5 (SD) vs. 1.6 ± 0.4 ng/mg tissue] (Figure 6). 

A key finding of the present study is that both Chol-siSTAT3 polyplexes and Chol-DsiSTAT3 polyplexes maintain the suppression of STAT3 mRNA in primary 4T1 breast tumors less than 24 h after IV administration of a single equimolar dose [0.50 mg Chol-DsiSTAT3/kg or 0.41 mg Chol-siSTAT3/kg] (Figure 3B). In contrast, Chol-DsiLUC polyplexes maintain the suppression of stably expressed LUC mRNA in primary 4T1-Luc breast tumors for ~48 h longer than Chol-siLUC polyplexes after daily IV administration of a higher dose over 3 days [2.5 mg/kg] [8]. One possible reason for discrepancies in the maintenance of mRNA suppression is that the DsiLUC sequence and/or corresponding Chol-DsiLUC polyplexes suppress LUC mRNA for a longer duration in 4T1-Luc cells within primary 4T1 tumors than the siLUC sequence and/or corresponding Chol-siLUC polyplexes. This is unlikely, however, given that, like the current DsiSTAT3 and siSTAT3 sequences (Figure 2A) and corresponding Chol-RNAi polyplexes (Figure 2B), electroporation with the DsiLUC or siLUC sequences and transfection with the corresponding Chol-RNAi polyplexes suppress LUC activity in 4T1-Luc cells with similar potency and kinetics over 72 h, respectively [8]. A second possibility is that Chol-DsiRNA polyplexes increase the maintenance of mRNA suppression in primary syngeneic murine breast tumors vs. Chol-siRNA polyplexes as the frequency of tumor-saturating doses is increased. This remains a possibility given that: (i). Chol-DsiSTAT3 polyplexes and Chol-siSTAT3 polyplexes suppress STAT3 mRNA in primary 4T1 tumors for a similar duration after IV administration of a single equimolar dose (Figure 3B) that does not saturate the primary 4T1 tumor [0.50 mg Chol-DsiSTAT3/kg and 0.41 mg Chol-siSTAT3/kg] (Figure 3A), (ii). Chol-DsiSTAT3 polyplexes suppress higher levels of total Stat3 protein [42% vs. 29%] (Figure 4C) and subsequent 4T1 tumor growth [rate-based T/C ratio of 8.6% vs. 11.5%] than Chol-siSTAT3 polyplexes after repeated IV administration every other day over 6 days (Figure 4A) at equimolar doses that saturate primary 4T1 breast tumors [2.0 mg Chol-siSTAT3/kg and 2.5 Chol-DsiSTAT3 mg/kg] (Figure 3A), and (iii). Chol-DsiLUC polyplexes suppress Luc activity in primary 4T1-Luc tumors 48 h longer than Chol-siLUC polyplexes after daily IV administration over 3 days [8] at doses that saturate primary 4T1 breast tumors [2.5 mg/kg] (Figure 3A). Thus, increases in the maintenance of mRNA suppression in solid breast tumors by Chol-DsiRNA polyplexes vs. Chol-siRNA polyplexes likely depend on both the targeted mRNA and the dosage regimen.

## 4. Materials and Methods

### 4.1. Polymer

Methoxy-poly(ethylene glycol)-*b*-poly(L-lysine hydrochloride) with a 5 kDa polyethylene glycol block (PEG[5K]) and a 30 poly-L-lysine block (PLL[30]) [PLL[30]-PEG[5K]; avg. MW 9900 Da] were obtained from Alamanda Polymers (Huntsville, AL, USA). The number of Lys residues within the PLL[30] block was ±10% (PLL[27] to PLL[33]), and the polydispersity index of the entire polymer was between 1 and 1.1.

### 4.2. RNAi Molecules

HPLC-purified siRNA and DsiRNA RNAi with indicated (UU) overhangs were obtained from GE Dharmacon: **(1) siCTRL** sense: 5′-CGU UAA UCG CGU AUA AUA C(UU)-3′, antisense: 5′-GUA UUA UAC GCG AUU AAC G(UU)-3′; **(2) DsiCTRL** sense: 5′-CGU UAA UCG GCU AUA AUA CGC GUA U-3′, antisense: 5′-AUA CGC GUA UUA UAC GCG AUU AAC G(UU)-3′; **(3) siSTAT3** sense: 5′-GGU CAA AUU UCC UGA GUU G(UU)-3′, antisense: 5′-CAA CUC AGG AAA UUU GAC C(UU)-3′; **(4) DsiSTAT3** sense: 5′-GGU CAA AUU UCC UGA GUU GAA UUA U-3′, antisense: 5′-AUA AUU CAA CUC AGG AAA UUU GAC C(UU)-3′; **(5)** **Chol-siCTRL:** siCTRL with 3′-cholesterol conjugated to the sense strand; **(6)** **Chol-siSTAT3:** siSTAT3 with 3′-cholesterol conjugated to the sense strand; **(7) Chol-DsiCTRL:** DsiSTAT3 with 3′-cholesterol conjugated to the sense strand; **(8) Chol-DsiSTAT3:** DsiSTAT3 with 3′-cholesterol conjugated to the sense strand. For in vitro studies, lyophilized RNAi molecules were resuspended in sterile, nuclease-free ddH_2_0 [100 μM] as directed (GE Dharmacon) and stored in aliquots [10 μL] at −80°C. For in vivo studies, lyophilized RNAi molecules were resuspended in the indicated buffer on the day of injection.

### 4.3. Cell Culture

Murine 4T1 breast cancer epithelial cells (CRL-2539, ATCC) were cultured and treated as described [2].

### 4.4. Electroporation of Murine 4T1 Cells

Murine 4T1 cells were electroporated with the indicated RNAi molecules and average STAT3 mRNA copies/ng total RNA were determined by ddPCR and compared as described [2].

### 4.5. Formation of Chol-RNAi Polyplexes

Chol-siRNA polyplexes and Chol-DsiRNA polyplexes were formed for transfection and in vivo studies as described [2].

### 4.6. Hydrodynamic Diameters and Zeta Potentials of Chol-RNAi Polyplexes

Average polyplex hydrodynamic diameters and zeta potentials were determined by nanoparticle tracking analysis and DLS, respectively, and compared as described [2]. 

### 4.7. Endotoxin Levels

Endotoxin levels of Chol-RNAi polyplexes were quantitated as described [2].

### 4.8. Transfection of Murine 4T1 Cells

Murine 4T1 cells were transfected with the indicated polyplexes and average STAT3 mRNA copies/ng total RNA were determined by RT-ddPCR and compared as described [2].

### 4.9. Quantitation of Murine STAT3 mRNA, Chol-siSTAT3, and Chol-DsiSTAT3 by Reverse Transcription-Droplet Digital™ PCR (RT-ddPCR)

Average STAT3 mRNA copies/mass total RNA (ng), mass Chol-siSTAT3 (ng)/mass tissue (mg), and mass Chol-DsiSTAT3 (ng)/mass tissue (mg) were determined by RT-ddPCR compared as described [2].

### 4.10. Potencies and Kinetics of STAT3 mRNA Suppression in Primary Murine 4T1 Breast Tumors by Chol-RNAi Polyplexes after IV Administration

All procedures were approved by the University of Nebraska Medical Center Institutional Animal Care and Use Committee. Primary 4T1 tumors were prepared in female BALB/c mice as described [2]. Average half-maximal ED_50_ and kinetics of Chol-siSTAT3 polyplexes and Chol-DsiSTAT3 polyplexes against murine STAT3 mRNA in primary murine 4T1 breast tumors after IV administration were determined by RT-ddPCR, compared, and calculated as described [2].

### 4.11. Tumor Growth Delay of Primary Murine 4T1 Breast Tumors

All procedures were approved by the University of Nebraska Medical Center Institutional Animal Care and Use Committee. Primary 4T1 tumors were prepared in female BALB/c mice as described [2]. Average rate-based T/C ratios and associated *p* values [17] against the growth of primary murine 4T1 breast tumors after repeated IV administration of vehicle, Chol-siSTAT3 polyplexes, and Chol-DsiSTAT3 polyplexes were calculated as described [2]. Average Stat3 protein levels on Day 8 (48 h after the final dose) were determined by western blot (Section 4.12) and compared as described [2].

### 4.12. Quantitation of Stat3 Protein in Primary 4T1 Tumors by Western Blot

Average ratios of Stat3/β-Actin protein band intensities ±SD (*n* = 2 measurements) were determined by western blot and imaging densitometry and compared as described [2].

### 4.13. Pharmacokinetics of Chol-RNAi after i.v. Administration

All procedures were approved by the University of Nebraska Medical Center Institutional Animal Care and Use Committee. Average plasma concentrations of Chol-siSTAT3 or Chol-DsiSTAT3 at each time point and subsequent pharmacokinetic parameters were determined and compared as described [2].

### 4.14. Distribution of Chol-RNAi Molecules in Tumors and Organs after i.v. Administration

All procedures were approved by the University of Nebraska Medical Center Institutional Animal Care and Use Committee. Average masses of Chol-siSTAT3 (ng)/mass of tissue (mg) or Chol-siSTAT3 (ng/)/mass of tissue (mg) were quantitated by RT-ddPCR (Section 4.9) and compared as described [2].

## 5. Conclusions

In summary, we found that Chol-siRNA polyplexes and Chol-DsiRNA polyplexes formed with PLL[30]-PEG[5K] increase potency and efficacy against STAT3 mRNA to the same extent in primary murine syngeneic breast tumors, although Chol-DsiRNA polyplexes are more physically stable in solution over 24 h and likely increase the duration of mRNA suppression in solid tumors as the frequency of tumor-saturating doses is increased. Thus, both Chol-siRNA polyplexes and Chol-DsiRNA polyplexes may be suitable clinical candidates for the RNAi therapy of breast cancer and other solid tumors.

## Figures and Tables

**Table 1:** Representative characteristics of Chol-siRNA polyplexes and Chol-DsiRNA polyplexes 30 min after complexation.

Polyplex	Endotoxin ^1^ EU/mg (±SD)	Drug Loading Wt% Chol-RNAi	Diameter ^2^ nm (±SD)	Zeta Potential ^3^ mV (±SD)
Chol-siCTRL Polyplexes (N/P 3)	0.122 (0.003)	25	25 (2)	8 (2)
Chol-DsiCTRL Polyplexes (N/P 1)	0.023 (0.002)	50	33 (2)	5.2 (0.7)

^1^ Determined by endochrome-K™ kit. ^2^ Determined by nanoparticle tracking analysis in 0.1 M HEPES, pH 7.4 and calculated from an average nonlinear fit of the lognormal distribution of polyplex diameters (*n* = 3 independent analyses) (Figure S2). ^3^ Determined by DLS in 0.1 M HEPES, pH 7.4 as an average of three independent analyses. Data for Chol-DsiCTRL polyplexes are taken from [2].

**Table 2:** Pharmacokinetic parameters of Chol-siSTAT3, Chol-DsiSTAT3, Chol-siSTAT3 polyplexes, and Chol-DsiSTAT3 polyplexes in healthy young female BALB/c mice after IV administration.

PK Parameter	Chol-siSTAT3 (±SD)	Chol-siSTAT3 Polyplexes (±SD)	Chol-DsiSTAT3 (±SD)	Chol-DsiSTAT3 Polyplexes (±SD)
C_0_ (μg/mL)	89 (65)	250 (123)	56 (64)	110 (7)
AUC_0–∞_ (h·μg/mL)	2.3 (0.9)	44 (2)	3 (1)	39 (13)
MRT_0–∞_ (h)	0.07 (0.03)	0.5 (0.5)	0.8 (0.9)	0.9 (0.4)

Average plasma concentration at time point = 0 (C_0_) and mean residence time (MRT_0–∞_) in healthy female BALB/c mice (4 to 6 weeks old) were determined by non-compartmental analysis (Phoenix WinNonlin) and average area under the curve (AUC_0–∞_) of Chol-DsiSTAT3 (*n* = 5 mice) was determined by the linear log trapezoidal rule of the respective PK profiles (Figure 5). Data for Chol-DsiSTAT3 and Chol-DsiSTAT3 polyplexes taken from [2].

## Data Availability

Data presented in this study are partially provided in the supplementary material or available on request from the corresponding author.

## Supplementary Materials

The following supporting information can be downloaded at https://www.mdpi.com/article/10.3390/ncrna8010008/s1, Figure S1. Complexation of Chol-siSTAT3 and Chol-DsiSTAT3 with PLL[30]-PEG[5K], Figure S2. Representative distributions of Chol-siCTRL polyplex and Chol-DsiCTRL polyplex diameters, Figure S3. Physical stabilities of Chol-siSTAT3 polyplexes and Chol-DsiSTAT3 polyplexes in solution over 24 h, Figure S4. Effect of inactive Chol-siCTRL and Chol-DsiCTRL polyplexes on the growth of primary 4T1 syngeneic breast tumors after multiple IV treatments, Table S1: Actual normalized STAT3 mRNA copy numbers for electroporation and transfection studies in 4T1 cells (Figure 2), Table S2: Actual STAT3 and HPRT1 mRNA copy numbers for potency and kinetics studies in primary 4T1 breast tumors (Figure 3).
