# Supplementary material for: Direct Comparison of Chol-siRNA Polyplexes and Chol-DsiRNA Polyplexes Targeting STAT3 in a Syngeneic Murine Model of TNBC
Source: Noncoding RNA. 2022 Jan 13;8(1):8. doi: 10.3390/ncrna8010008 (PMC8788547; doi:10.3390/ncrna8010008)
Supplement: Supplementary file 1 [file ncrna-08-00008-s001.zip › ncrna-1396620-supplementary.pdf]

non-coding RNA

MDPI

Article

Direct Comparison of Chol-siRNA Polyplexes and Chol-DsiRNA Polyplexes Targeting STAT3 in a Syngeneic Murine Model of TNBC

Zhen Ye [1], Mai Mohamed Abdelmoaty [1,2], Stephen M. Curran [1], Shetty Ravi Dyavar [3,†], Devendra Kumar [1], Yazen Alnouti [1,4], Don W. Coulter [5], Anthony T. Podany [3], Rakesh K. Singh [4,6] and Joseph A. Vetro [1,4,*]

1 Department of Pharmaceutical Sciences, College of Pharmacy, University of Nebraska Medical Center, Omaha, NE 68198, USA; yezhen1@gmail.com (Z.Y.); mai.mostafa@unmc.edu (M.M.A.); smcurran@unmc.edu (S.M.C.); devendra.kumar@unmc.edu (D.K.); yalnouti@unmc.edu (Y.A.)
2 Therapeutic Chemistry Department, Pharmaceutical and Drug Industries Research Division, National Research Centre, Giza 12622, Egypt
3 Department of Pharmacy Practice, College of Pharmacy, University of Nebraska Medical Center, Omaha, NE 68198, USA; dyavarsravi@gmail.com (S.R.D.); apodany@unmc.edu (A.T.P.)
4 Center for Drug Delivery and Nanomedicine, University of Nebraska Medical Center, Omaha, NE 68198, USA
5 Department of Pediatrics, Division of Pediatric Hematology/Oncology, University of Nebraska Medical Center, Omaha, NE 68198, USA; dwcoulter@unmc.edu (D.W.C.)
6 Department of Pathology and Microbiology, University of Nebraska Medical Center, Omaha, NE 68198, USA; rsingh@unmc.edu
* Correspondence: jvetro@unmc.edu; Tel.: +1-402-559-9359
† Current address: Adicet Bio, Inc., 200 Constitution Dr, Menlo Park, CA 94025, USA.

Citation: Ye, Z.; Abdelmoaty, M.M.; Curran, S.M.; Dyavar, S.R.; Kumar, D.; Alnouti, Y.; Coulter, D.W.; Podany, A.T.; Singh, R.K.; Vetro, J.A. Direct Comparison of Chol-siRNA Polyplexes and Chol-DsiRNA Polyplexes Targeting STAT3 in a Syngeneic Murine Model of TNBC. *Non-coding RNA* **2022**, *8*, 8. https://doi.org/10.3390/ncrna8010008

Academic Editors: Sébastien Pfeffer and Anil K. Sood

Received: 10 September 2021
Accepted: 10 January 2022
Published: 13 January 2022

Publisher's Note: MDPI stays neutral with regard to jurisdictional claims in published maps and institutional affiliations.

Copyright: © 2022 by the authors. Licensee MDPI, Basel, Switzerland. This article is an open access article distributed under the terms and conditions of the Creative Commons Attribution (CC BY) license (https://creativecommons.org/licenses/by/4.0/).

Supplementary Materials

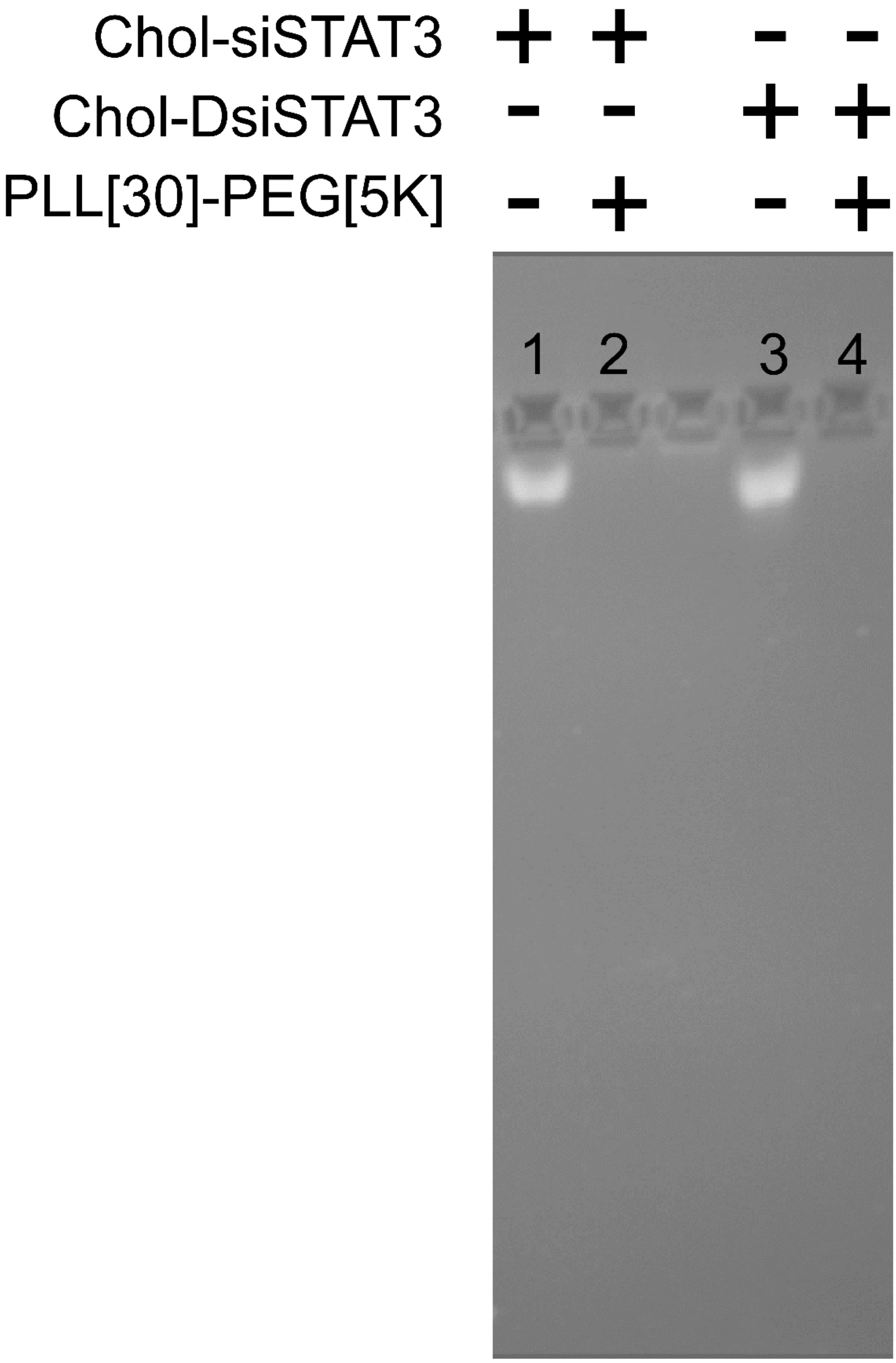

Figure S1. Complexation of Chol-siSTAT3 and Chol-DsiSTAT3 with PLL[30]-PEG[5K]. (A) Chol-siSTAT3 or Chol-DsiSTAT3 was incubated with PLL[30]-PEG[5K] in 0.1 M HEPES [pH 7.4] for 30 minutes at room temperature. Chol-siSTAT3 alone (Lane 1), Chol-siSTAT3 Polyplexes [N/P 3] (Lane 2), Chol-DsiSTAT3 alone (Lane 3), and Chol-DsiSTAT3 Polyplexes [N/P 1] (Lane 4) (90 ng Chol-RNAi each) were then separated on a 4% TBE agarose gel (120 V, 30 min) that was post-stained with SYBR Green II for subsequent imaging. Data are representative of at least two independent experiments.

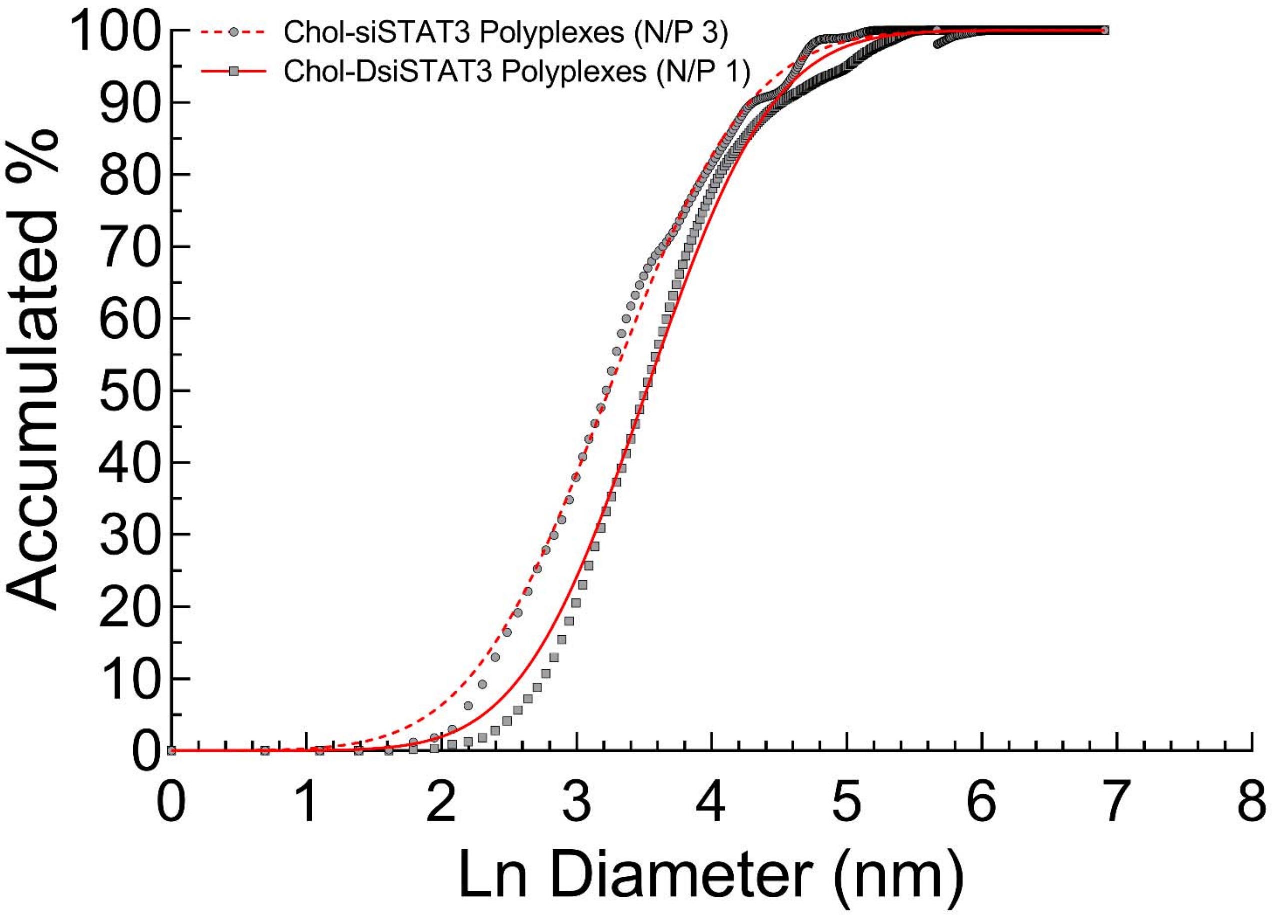

Figure S2. Representative Distributions of Chol-siCTRL Polyplex and Chol-DsiCTRL Polyplex Diameters. Average diameters of Chol-siCTRL Polyplexes and Chol-DsiCTRL Polyplexes ± SD (n = 3 independent sample measurements) were determined in 0.1 M HEPES, pH 7.4 by nanoparticle tracking analysis (NTA). A plot of accumulated percent of total Chol-siRNA Polyplexes (grey circles) or Chol-DsiRNA Polyplexes (grey squares) at each diameter (y-axis) vs. ln diameter (x-axis) was then fit against a cumulative Gaussian (percent) model using GraphPad Prism 9 to determine a best-fit mean (dashed red line for Chol-siCTRL Polyplexes and solid red line for Chol-DsiCTRL Polyplexes) and standard deviation from the lognormal curve. Data for Chol-DsiCTRL Polyplexes taken from [2].

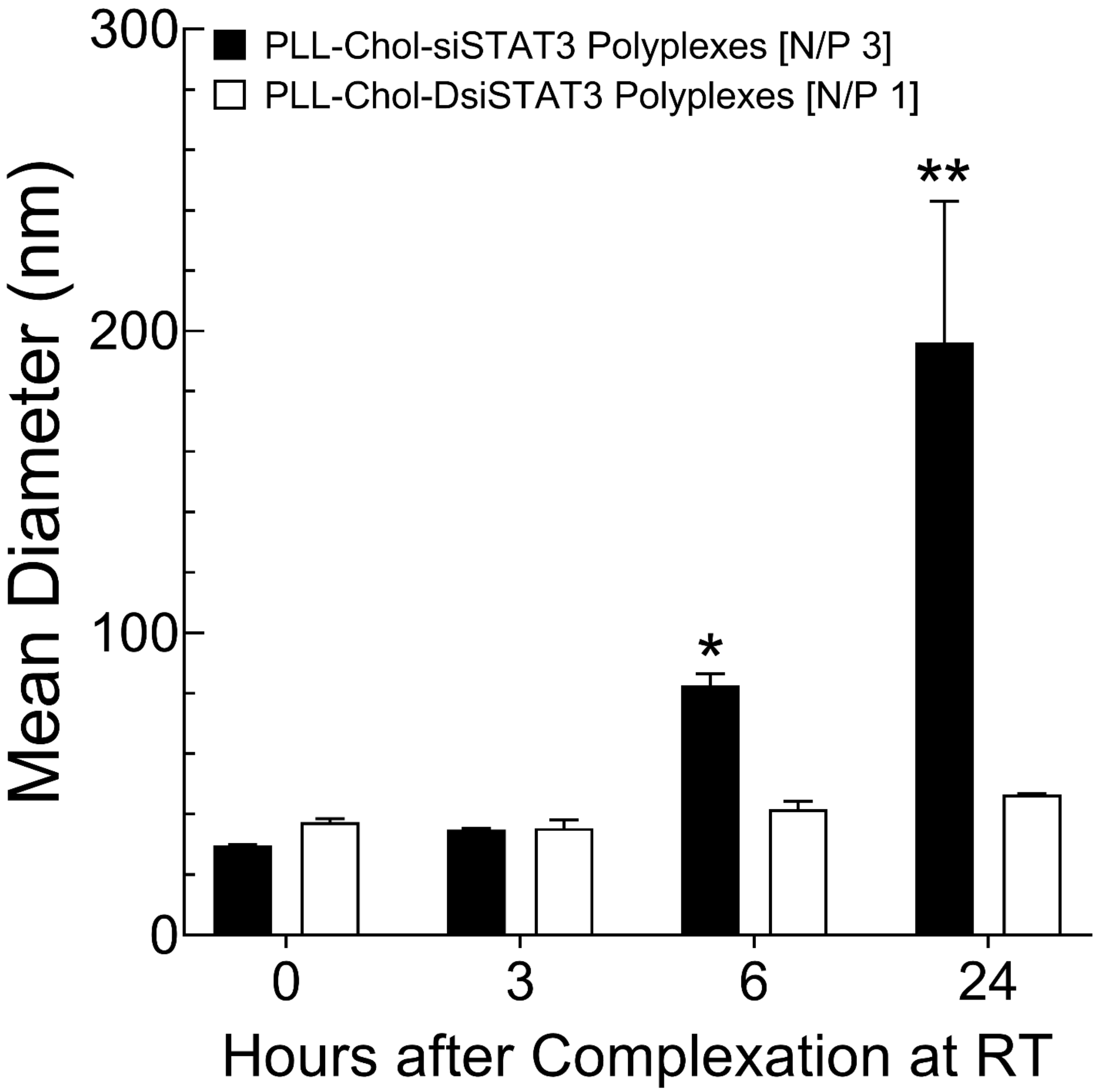

Figure S3. Physical stabilities of Chol-siSTAT3 Polyplexes and Chol-DsiSTAT3 Polyplexes in solution over 24 hours. Average hydrodynamic diameters of Chol-siSTAT3 Polyplexes and Chol-DsiSTAT3 Polyplexes ± SD ($n = 3$ independent sample measurements) in 0.1 M HEPES, pH 7.4 were determined by nanoparticle tracking analysis (NTA) (**Figure S2**) after incubation at room temperature for the indicated time points and compared vs. time point 0 by Ordinary one-way ANOVA with Dunnet's post-test where *$p < 0.05$ and **$p < 0.01$.

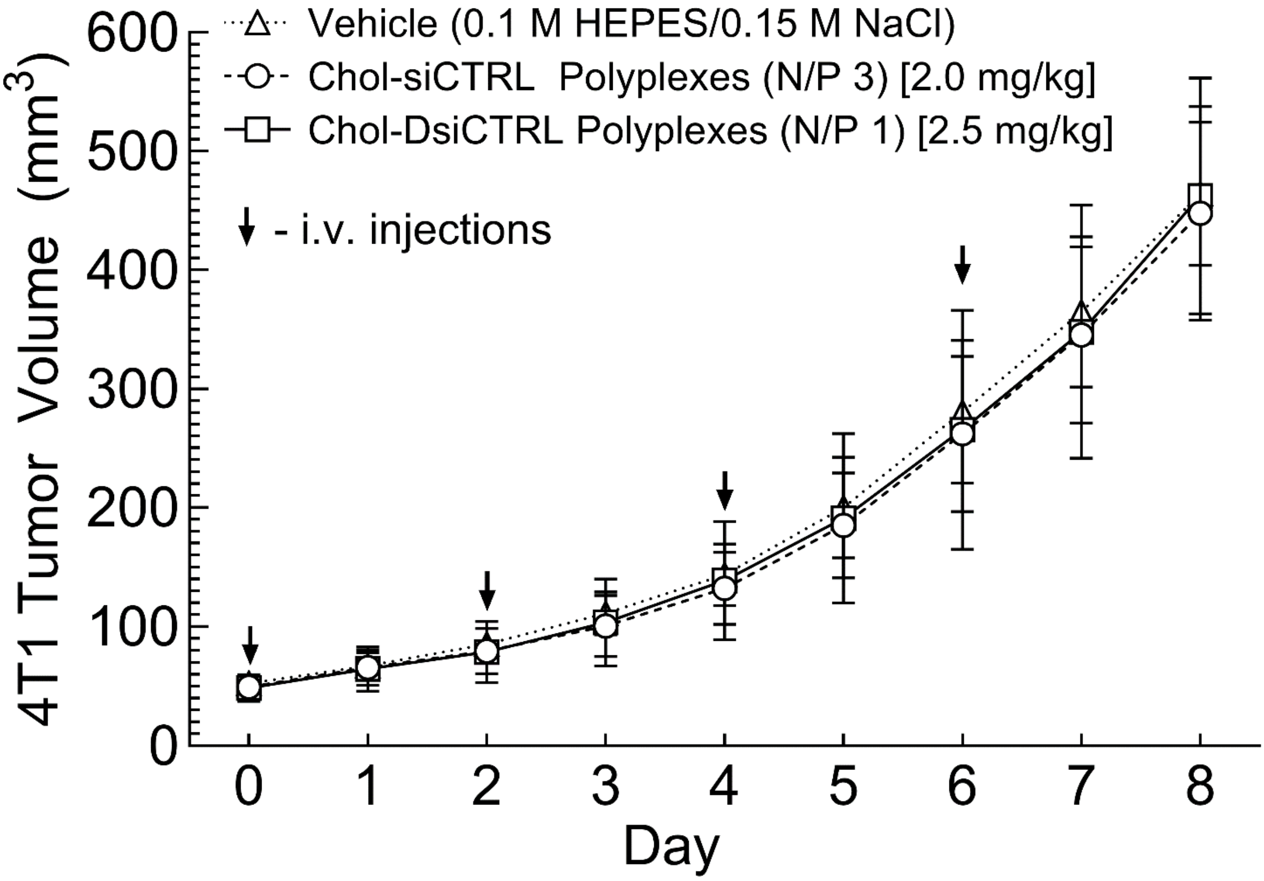

Figure S4. Effect of inactive Chol-siCTRL and Chol-DsiCTRL Polyplexes on the growth of primary 4T1 syngeneic breast tumors after multiple i.v. treatments. Vehicle alone (white triangles) or vehicle containing an equimolar does of inactive Chol-siCTRL (white circles) or inactive Chol-DsiCTRL (white squares) complexed with PLL[30]-PEG[5K] at the indicated N/P ratio was injected into the tail veins of female BALB/c mice (black arrows) bearing a single subcutaneous 4T1 breast tumor (30 to 50 mm^3). Average daily tumor volumes ±SD (n = 5 mice) were then determined by 3D surface scanning and compared at each time point by Multiple t-tests. Data for Vehicle and Chol-DsiCTRL taken from [2].

Table S1: Actual normalized STAT3 mRNA copy numbers for electroporation and transfection studies in 4T1 cells (Figure 2).

Treatment	Hours Post-Treatment	STAT3 mRNA copies/ ng cDNA (± SD)[a]
Electroporation		
Electroporation only[c]	24	353 (29)
"	48	435 (4)
"	72	432 (20)
siCTRL	24	333 (19)
siSTAT3	24	70 (1)
"	48	263 (7)
"	72	390 (12)
DsiCTRL[c]	24	325 (13)
DsiSTAT3[c]	24	68 (1)
"	48	248 (14)
"	72	392 (7)
Transfection		
Untreated[c]	24	371(18)
"	48	472 (10)
"	72	520 (17)
Chol-siCTRL Polyplexes	24	328 (15)
Chol-siSTAT3 Polyplexes	24	155 (7)
"	48	287 (12)
"	72	490 (14)
Chol-DsiCTRL Polyplexes[c]	24	325 (13)
Chol-DsiSTAT3 Polyplexes[c]	24	134 (15)
"	48	298 (9)
"	72	479 (14)

[a] Average STAT3 mRNA copies/ng cDNA ± propagated SD (n = 2 ddPCR replicates from two independent electroporations) determined by RT-ddPCR. [c] Data from [2].

Table S2: Actual STAT3 and HPRT1 mRNA copy numbers for potency and kinetics studies in primary 4T1 breast tumors (Figure 3).

Treatment	Hours Post-treatment	STAT3 mRNA copies/ ng cDNA (± SD)[a]	HPRT1 mRNA copies/ ng cDNA (± SD)[a]	STAT3/HPRT1 Ratio (± SD)[b]
Potency (ED_{50})				
Vehicle (Chol-siSTAT3 Polyplexes)	48	2116 (157)	1119 (78)	1.9 (0.2)
Chol-siCTRL Polyplexes [4.0 mg/kg]	48	2259 (137)	1165 (154)	1.9 (0.3)
Chol-siSTAT3 Polyplexes (0.24 mg/kg]	48	1309 (86)	865 (47)	1.5 (0.1)
Chol-siSTAT3 Polyplexes [0.48 mg/kg]	48	1396 (155)	1098 (115)	1.3 (0.2)
Chol-siSTAT3 Polyplexes [0.8 mg/kg]	48	1327 (71)	1170 (70)	1.1 (0.1)
Chol-siSTAT3 Polyplexes [2.0 mg/kg]	48	1505 (109)	1433 (95)	1.0 (0.1)
Chol-siSTAT3 Polyplexes [4.0 mg/kg]	48	1278 (90)	1268 (100)	1.0 (0.1)
Vehicle (Chol-DsiSTAT3 Polyplexes)[c]	48	2449 (69)	1270 (52)	1.9 (0.1)
Chol-DsiCTRL Polyplexes [5.0 mg/kg][c]	48	2910 (330)	1447 (197)	2.0 (0.4)
Chol-DsiSTAT3 Polyplexes [0.3 mg/kg][c]	48	2205 (78)	1453 (49)	1.5 (0.1)
Chol-DsiSTAT3 Polyplexes [0.6 mg/kg][c]	48	2181 (118)	1734 (92)	1.3 (0.1)
Chol-DsiSTAT3 Polyplexes [1.0 mg/kg][c]	48	1832 (104)	1582 (65)	1.2 (0.1)
Chol-DsiSTAT3 Polyplexes [2.5 mg/kg][c]	48	2258 (94)	2055 (124)	1.1 (0.1)
Chol-DsiSTAT3 Polyplexes [5.0 mg/kg][c]	48	2125 (80)	2053 (109)	1.0 (0.1)
Kinetics				
Vehicle[c]	24	2844 (316)	1554 (213)	1.8 (0.3)
"	48	3233 (863)	1701 (444)	1.9 (0.7)
"	72	2571 (419)	1334 (183)	1.9 (0.4)
"	96	2831 (328)	1514 (156)	1.9 (0.3)
Chol-siSTAT3 Polyplexes [0.41 mg/kg]	24	2911 (263)	1788 (141)	1.6 (0.2)
"	48	2202 (164)	2009 (125)	1.1 (0.1)
"	72	3016 (455)	1803 (257)	1.7 (0.3)
"	96	2739 (244)	1585 (167)	1.7 (0.2)
Chol-DsiSTAT3 Polyplexes [0.5 mg/kg][c]	24	2865 (169)	1742 (83)	1.6 (0.1)
"	48	1537 (126)	1409 (121)	1.1 (0.1)
"	72	2724 (386)	1593 (221)	1.7 (0.3)

"	96	2666 (248)	1506 (138)	1.8 (0.2)

[a] Average mRNA copies/ng cDNA ± propagated SD in primary 4T1 breast tumors (*n* = 3 ddPCR replicates from 5 mice) determined by RT-ddPCR. [b] Ratio of STAT3 mRNA copy number / HPRT1 copy number ± propagated SD. [c] Data from [2].
